# Timing of Onset of Garadacimab for Preventing Hereditary Angioedema Attacks

**DOI:** 10.1111/cea.14568

**Published:** 2024-10-01

**Authors:** Petra Staubach, Raffi Tachdjian, H. Henry Li, Roman Hakl, Emel Aygören‐Pürsün, Lolis Wieman, John‐Philip Lawo, Timothy J. Craig

**Affiliations:** ^1^ Department of Dermatology and Allergy University Medical Center Mainz Mainz Germany; ^2^ Division of Allergy and Clinical Immunology, David Geffen School of Medicine University of California, Los Angeles Los Angeles California USA; ^3^ Institute for Asthma and Allergy Chevy Chase Maryland USA; ^4^ Department of Clinical Immunology and Allergology, St. Anne's University Hospital and Faculty of Medicine Masaryk University Brno Czech Republic; ^5^ Department for Children and Adolescents, University Hospital Frankfurt Goethe University Frankfurt Frankfurt Germany; ^6^ CSL Behring King of Prussia Pennsylvania USA; ^7^ CSL Innovation GmbH Marburg Germany; ^8^ Allergy and Immunology, Tenured Professor of Medicine Pediatrics and Biomedical Sciences Hershey Pennsylvania USA; ^9^ Vinmec International Hospital Times City Hanoi Vietnam

**Keywords:** attack free, early onset, garadacimab, hereditary angioedema, long‐term prophylaxis

**Summary**

- Garadacimab provided consistent HAE attack prevention in manufacturer‐funded post hoc pivotal Phase 3 data analysis.
- Protection from HAE attacks occurred by Week 1 after first administration.

**Abbreviations**
C1‐INH: C1‐esterase inhibitor
CI: confidence interval
EAACI: European Academy of Allergy and Clinical Immunology
FXII: factor XII
HAE: hereditary angioedema
LTP: long‐term prophylactic
WAO: World Allergy Organization

To the editor,

Hereditary angioedema (HAE) is a rare, autosomal dominant disease characterised by recurrent, unpredictable, painful, debilitating and potentially life‐threatening attacks [1, 2, 3]. HAE imparts a substantial disease burden that impacts daily activities and extends beyond symptoms directly attributable to HAE attacks, encompassing mental health (anxiety and depression) and psychosocial impacts associated with unpredictable attack recurrence [1, 3].

Per World Allergy Organization (WAO)/European Academy of Allergy and Clinical Immunology (EAACI) guidelines, the goal of HAE treatment is complete disease control and normalisation of patients' lives [2]. This can only be achieved with effective long‐term prophylactic (LTP) therapy [2]. Early onset of efficacy and durability of protection are critical attributes of LTP therapies to optimise HAE disease control and establish clinician and patient confidence in the treatment. Despite the availability of approved LTP therapies, there is still an unmet need for treatments with a rapid onset and improved durability of protection against HAE attacks [2, 4].

Activated factor XII (FXIIa) is the principal initiator of the kallikrein–kinin system, leading to production of bradykinin, the key inflammatory mediator responsible for vasodilation and vascular permeability [2, 5, 6]. C1 inhibitor (C1INH) regulates FXIIa in healthy individuals [5, 6]. In HAE, most patients have C1INH deficiency (HAE‐C1INH‐Type1) or dysfunction (HAE‐C1INH‐Type2), leading to uncontrolled activation of FXII. The subsequent dysregulation of the kallikrein–kinin system results in overproduction of bradykinin, ultimately leading to HAE attacks [2, 5, 6].

Garadacimab is a first‐in‐class, fully human, potent, anti‐activated FXII monoclonal antibody under clinical evaluation as an LTP therapy for HAE attacks [7, 8]. Garadacimab has high affinity and specificity for FXIIa, with in vitro data demonstrating decreased bradykinin production [8]. In the 6‐month pivotal Phase 3 (VANGUARD) study (NCT04656418), patients aged ≥ 12 years with HAE with C1INH deficiency or dysfunction and an average attack rate of ≥ 3 attacks in the 3 months preceding study initiation were randomised (3:2) to receive garadacimab 200 mg subcutaneous once monthly after an initial 400 mg loading dose (*n* = 39) or volume‐matched placebo (*n* = 25). Garadacimab significantly reduced monthly number of attacks versus placebo (mean: 0.27 vs. 2.01, respectively; *p* < 0.0001 and median [interquartile range] 0.00 [0.00–0.31] vs. 1.35 [1.00–3.20], respectively). Throughout the study, 62% of patients treated with garadacimab remained attack free, demonstrating durable protection against HAE attacks [7].

In this post hoc analysis of the pivotal Phase 3 (VANGUARD) study, the onset of protection against HAE attacks was assessed based on actual numbers of attacks (i.e., non‐extrapolated data). The time‐normalised mean monthly number of attacks and percentage of attack‐free patients were calculated at weekly intervals for the first 4 weeks and monthly intervals for the rest of the 6‐month study duration. Treatment with garadacimab reduced the mean monthly number of attacks as early as Week 1 after the first administration versus placebo (Figure 1A). At Week 1, the time‐normalised mean (95% confidence interval [CI]) monthly number of attacks in the garadacimab group was 0.11 (−0.11 to 0.34) versus 3.07 (2.41–3.73) at run‐in; in the placebo group, it was 1.81 (0.74–2.88) versus 2.52 (2.13–2.91) at run‐in. The mean monthly number of attacks was reduced with garadacimab versus run‐in and versus placebo through Month 6 (study end) (Figure 1A).

**FIGURE 1:**
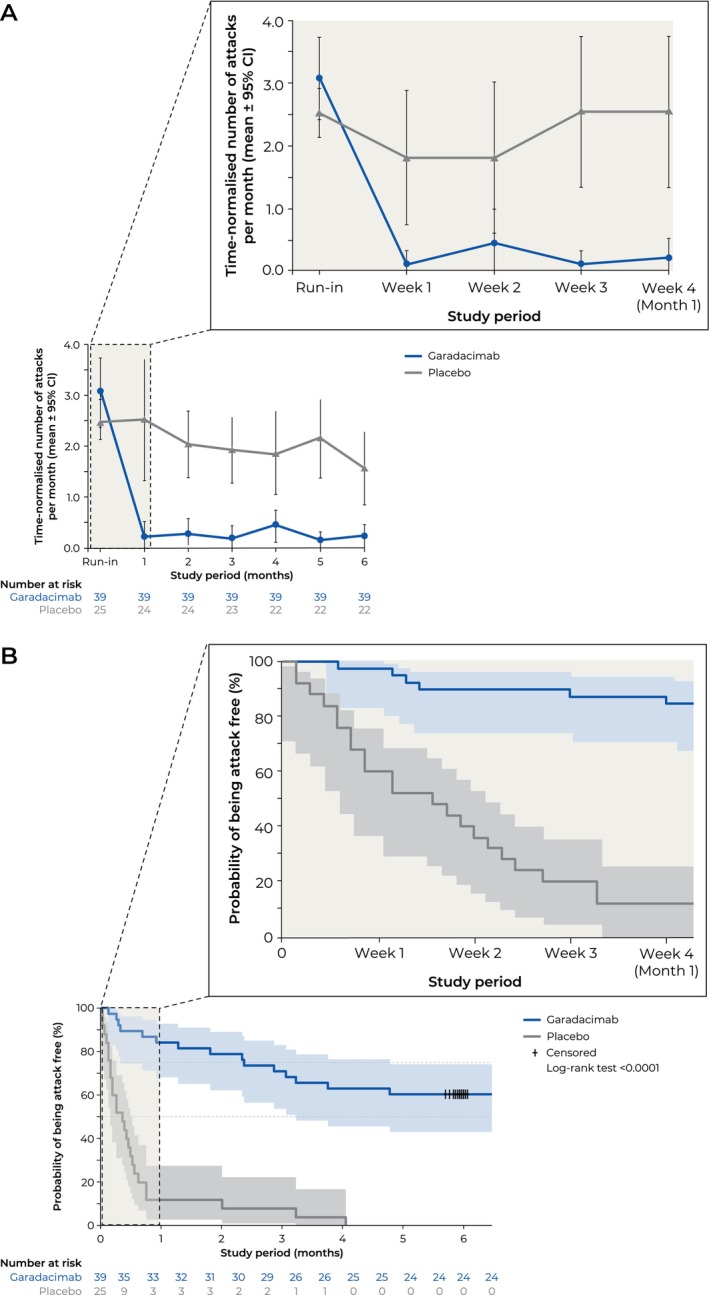
Early onset of protection against HAE attacks in the pivotal Phase 3 VANGUARD study. Garadacimab reduced HAE attack rate versus placebo from Week 1 through Month 6. 95% CIs are represented by error bars (A) and shaded areas (B). Panel B has been previously published; copyright received under the Creative Commons license [7]. CI, confidence interval; HAE, hereditary angioedema.

Garadacimab reduced the mean (95% CI) monthly number of attacks versus run‐in by 96.3% (88.9–103.8), 85.9% (69.7–102.0), 96.0% (87.8–104.1) and 88.0% (68.2–107.8) at Weeks 1, 2, 3 and 4, respectively, versus 26.1% (−17.0 to 69.2), 39.3% (−4.2 to 82.7), −5.0% (−62.8 to 52.8) and −12.7% (−86.4 to 61.0) for placebo, respectively.

Patients receiving garadacimab had a higher probability of remaining attack free than those receiving placebo in any given week, starting as early as Week 1 (Figure 1B). At Weeks 1, 2, 3 and 4, 97.4%, 92.3%, 97.4% and 94.9% of patients remained attack free in the garadacimab group, respectively. In the placebo group, 62.5%, 66.7%, 50.0% and 50.0% of patients were attack free at Weeks 1, 2, 3 and 4, respectively, reflecting the variability of attack occurrence, which is characteristic of HAE (Figure 1B) [1, 2]. Due to this variability in attack occurrence, the weekly attack rates described in this analysis should be interpreted with caution [1, 2].

The proportion of patients who were attack free with garadacimab was sustained for any given month through Month 6, ranging from 76.9% to 89.7% with garadacimab versus 9.1%–36.4% with placebo [7]. Cumulatively, patients receiving garadacimab had a higher probability of remaining attack free than those receiving placebo from the first administration through Month 6 (Figure 1B). No patients receiving placebo were attack free through Month 6.

Published pharmacokinetic data from the pivotal Phase 3 (VANGUARD) study showed that garadacimab reached steady‐state plasma concentrations after the first administration, which remained at steady state throughout the treatment period [7]. This supports the mechanistic basis for FXIIa inhibition with garadacimab, driving the observed early onset of protection against HAE attacks.

This post hoc analysis demonstrated that treatment with garadacimab results in early and durable protection against HAE attacks from Week 1 sustained to Month 6. This contributes substantially towards the achievement of the primary goal of HAE treatment: complete disease control and normalisation of patients' lives, per the latest WAO/EAACI guidelines [2]. Protection against HAE attacks begins after the first administration of garadacimab, allowing clinicians to be confident in its treatment effect as early as Week 1 after treatment initiation.

These data support previously published evidence for garadacimab as an LTP therapy for HAE, based on early and durable protection against HAE attacks, and a favourable safety and tolerability profile [7, 9].

## Author Contributions

P.S., R.T., H.H.L., R.H. and E.A.‐P. contributed to data interpretation. T.J.C. and J.‐P.L. contributed to the overall study design and design of post hoc analysis. L.W. contributed to the overall study design and review of the study manuscript.

## Conflicts of Interest

Petra Staubach has received honoraria, research funding and travel grants from and/or has served as a consultant for and/or has participated in advisory boards for BioCryst, CSL Behring, KalVista, Octapharma, Pharming, Pharvaris, Shire and Takeda. Raffi Tachdjian has received consultancy/research support from Allakos, BioCryst, CSL Behring, Ionis, KalVista, Pharming, Pharvaris and Takeda, and speaker fees from AstraZeneca, BioCryst, CSL Behring, Grifols, GSK, Pharming, Sanofi/Regeneron and Takeda. H. Henry Li has received speaker fees for BioCryst, CSL Behring, Pharming and Takeda, and research and consultancy grants from BioCryst, BioMarin, CSL Behring, Ionis, Pharming, Pharvaris and Takeda. Roman Hakl has received speaking/consultancy fees and travel grants from and/or has participated in advisory boards for CSL Behring, Pharming, Shire and Takeda, and has served as a Principal Investigator for clinical trials sponsored by BioCryst, CSL Behring, KalVista, Pharming and Pharvaris. Emel Aygören‐Pürsün has received honoraria as a speaker/advisor for and/or grant/clinical trial investigator support from Astria Therapeutics, BioCryst, BioMarin, Centogene, CSL Behring, Intellia Therapeutics, KalVista, Pharming, Pharvaris and Takeda/Shire. Lolis Wieman is a full‐time employee of CSL Behring LLC and shareholder of CSL Limited. John‐Philip Lawo is a full‐time employee of CSL Behring Innovation GmbH and shareholder of CSL Limited. Timothy J. Craig is a speaker for CSL Behring, Grifols, KalVista and Takeda; has received research and consultancy grants from BioCryst, BioMarin, CSL Behring, Grifols, Ionis, KalVista, Pharvaris, Astria and Takeda; is on the Medical Advisory Board for the US Hereditary Angioedema Association, Director of ACARE Angioedema Center at Penn State University, Hershey, PA, USA.

## Data Availability

CSL Behring will consider requests to share Individual Patient Data (IPD) from CSL Behring‐sponsored studies with external bona fide, qualified scientific and medical researchers on a case‐by‐case basis. When appropriate, IPD will generally be shared after review by major regulatory authorities (i.e., Food and Drug Administration, European Medicines Agency) is complete and the primary publication is available. Proposed research should seek to answer a previously unanswered important medical or scientific question. Requests should reflect those important questions. Applicable country‐specific privacy and other laws and regulations will be considered and may prevent sharing of IPD. A research proposal detailing the use of the IPD will be reviewed by an internal CSL Behring review committee. If the request is approved, and the researcher agrees to the applicable terms and conditions in a data sharing agreement, IPD that have been appropriately anonymised will be made available. Supporting documents including the study protocol and statistical analysis plan will also be provided to the researcher. For information on the process and requirements for submitting a voluntary data‐sharing request for IPD, please contact CSL Behring at clinicaltrials@cslbehring.com.
